# Phenotypes of a family with XLH with a novel *PHEX* mutation

**DOI:** 10.1038/s41439-020-0095-1

**Published:** 2020-03-31

**Authors:** Akiko Yamamoto, Toshiro Nakamura, Yasuhisa Ohata, Takuo Kubota, Keiichi Ozono

**Affiliations:** 1 Department of Pediatrics, Kumamoto Chuo Hospital, Kumamoto, Japan; 2 Department of Pediatrics, Osaka University Graduate School of Medicine, Osaka, Japan

**Keywords:** Parathyroid diseases, Parathyroid diseases

## Abstract

X-linked hypophosphatemia (XLH) is the most common form of heritable hypophosphatemic rickets. We encountered a 4-year-old boy with a novel variant in the phosphate-regulating neutral endopeptidase homolog X-linked (*PHEX*) gene who presented with a short stature, genu valgum, and scaphocephaly. The same mutation was identified in his mother and sister; however, the patient presented with a more severe case.

X-linked hypophosphatemia (XLH) is an X-linked dominant disorder and the most common form of heritable rickets, with a case rate estimate of ~1 case per 20,000 live births^1^. Inactive mutations in *PHEX*, which is located in Xp22.1-22.2, have been implicated in the pathogenesis of XLH. More than 200 different mutations in *PHEX* have been identified to date^2^. XLH is characterized by rickets accompanied by bone deformities, a short stature, dental anomalies, bone pain, hearing difficulties, enthesopathy, and muscular dysfunction^3^. Patients with XLH have hypophosphatemia with low levels of renal phosphate reabsorption, normal serum calcium levels, elevated serum alkaline phosphatase activities, normal or increased parathyroid hormone levels, normal or increased 1,25-dihydroxyvitamin D3 levels, and elevated FGF23 levels^4^. Marked variations in XLH phenotypes have been reported. We herein describe a boy and his family who had XLH and a novel splice-site mutation in *PHEX*.

A boy aged 4 years and 11 months presented with a short stature to the endocrinology unit of our hospital. He was born after a normal pregnancy, with a birth weight of 3130 g and body length of 48 cm. He had an elder brother as well as a younger brother and sister. His mother and grandmother both had a short stature and genu valgum, whereas his elder and younger brothers did not have a short stature (Fig. 1(a)).

**Fig. 1:**
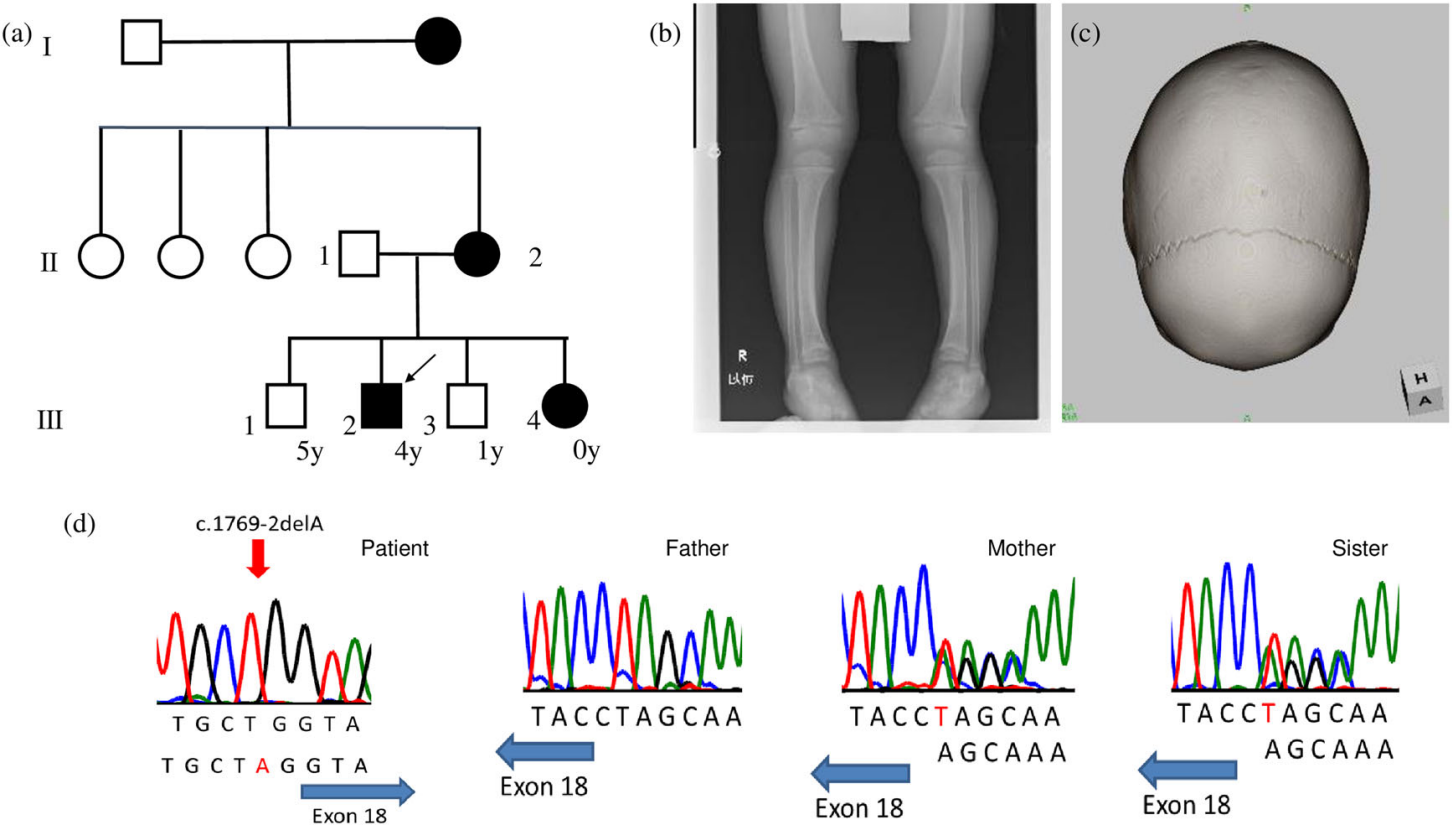
Clinical characteristics and genetic findings in the patient. **a** A family tree: the solid square indicates the index case. Solid circles indicate family members who developed rickets. Open symbols indicate healthy members. **b** A radiological examination showing fraying and cupping of the metaphysis and bilateral leg deformation. **c** Three-dimensional CT showing skull bone window reconstruction. The complete closure of sagittal sutures. **d** Gene analysis. A novel splice acceptor site mutation, c.1769-2delA, was identified in *PHEX*. The same heterozygous mutation was detected in the mother and sister of the patient. No mutations were found in the father.

At the time of his visit, his height was 95.4 cm (−2.51 standard deviation score (SDS)), weight was 14.8 kg (−1.13 SDS), and growth rate was 5.2 cm per year (−1.40 SDS), and genu valgum and scaphocephaly were both evident. He did not have any dental issues or other symptoms.

His serum calcium level was 9.71 mg/dl (normal range: 8.8–10.3 mg/dl), phosphate level was 2.4 mg/dl (3.8–5.9 mg/dl), alkaline phosphatase (ALP) level was 1175 IU/L (420–1200 mg/dl), intact parathyroid hormone (PTH) (ECLIA) level was 73 pg/ml (10–65 pg/ml), and 25(OH) vitamin D (RIA) level was 17 ng/ml (>30 ng/ml). He had a low % tubular reabsorption of phosphorus (TRP) value (89.3%) and a high intact FGF23 (ELISA) level (73 pg/ml) (16–69 pg/ml). Bilateral femoral and tibial bowing with fraying and cupping of the metaphysis were noted on the X-ray images (Fig. 1b). Brain CT scans showed craniosynostosis (Fig. 1c) and a mild Chiari malformation.

Since XLH was suspected, a genetic analysis was performed, which was approved by the Institutional Review Boards of the participating institutions. Written informed consent was obtained from the study participants, including their consent to participate and permission to publish the findings obtained. Genomic DNA was extracted from whole blood samples using a magLEAD Consumable Kit (Precision System Science Co., Ltd., Chiba, Japan). The amplicons generated by PCR with the designed primers were Sanger sequenced using a 3730 DNA analyzer (Applied Biosystems, Foster City, CA, USA), which revealed a novel *PHEX* mutation in intron 17, NM_000444.6: c.1769-2delA. We analyzed this novel mutation by Human Splicing Finder 3.1 (http://www.umd.be/HSF3/index.html), and the results obtained suggested that this mutation led to a broken WT acceptor site and affected splicing. The mutation detected in this patient was not found in the Human Gene Mutation Database (http://www.hgmd.cf.ac.uk/ac/index.php) or Genome Aggregation Database (http://gnomad.broadinstitute.org).

The diagnosis of XLH was confirmed by a genetic analysis, and therapy comprising phosphate 55 mg/kg/day and alfacalcidol 35 ng/kg/day was initiated. DNA samples obtained from the patient’s younger sister, mother, and father were also subjected to a genetic analysis. No abnormalities were detected in *PHEX* in the father, whereas the mother and sister had the same mutation. His mother and sister both had hypophosphatemia, elevated ALP enzyme activities, and high intact FGF23 levels (Table 1, Fig. 1d). His mother also had a short stature (148 cm) and genu valgum, whereas his sister only presented with mild genu valgum (her height was −1.78 SD). Although we were unable to genetically analyze the DNA from his grandmother, she also had a short stature, genu valgum, and bone pain.

**Table 1:** Laboratory data (%TRP (tubular reabsorption of phosphorus) = 1−(urine phosphorus × serum creatinine)/(serum phosphorus × urine creatinine)).

Family No.	Age	Height (cm)	Body weight (kg)	Calcium (mg/dl)	Phosphorus (mg/dl)	Alkaline phosphatase (U/L)	intact PTH (U/L)	intact FGF23 (pg/ml)	1,25(OH)_2_ vitamin D (pg/ml)	25(OH) vitamin D (ng/ml)	%TRP (%)
Patient III-2	4y 11m	95.4 (−2.51 SD)	14.8	9.71	2.4	1175	73	73	61.2	17	89.3
Sister III-4	0y 6m	62.1 (−1.78 SD)	6.635	9.70	4.1	1408	39	93	82.9	26	87.7
Mother II-2	34y	148 (−1.91 SD)	48	8.9	2.2	265	69	87	85.0	16.4	84.2
Father II-1	34y	169 (−0.31 SD)	78	10.1	3.6	269	33	41	108	22.3	92.4

We herein described a novel variant mutation in the splice site of intron 17 in *PHEX* of a boy and his family with XLH. XLH is a dominant disorder and the most common form of heritable rickets, and inactive mutations in *PHEX* have been implicated in the pathogenesis of XLH. In accordance with the present results, splice-site mutations have been shown to be responsible for 17% of all reported *PHEX* mutations^5^. Other mutations have also been reported in the splice acceptor site of intron 17^2,6,7^. Hue Yue et al. described a case of a 16-year-old boy with a *PHEX* gene mutation in the splice acceptor site of intron 17 (c.1768 + 2 T > G). This was a sporadic case that was characterized by leg bowing, hip pain, and growth retardation. In contrast, the present case had a familial mutation in *PHEX* along with a short stature, genu valgum, and scaphocephaly. Scaphocephaly is a form of craniosynostosis, a cranial malformation caused by premature fusing of the sagittal suture, and may develop in infants as young as 1 year of age^3,8^. Craniosynostosis occurs in ~60% of children with XLH^9^. Between 25% and 50% of children with XLH also have a Chiari type 1 malformation, which causes the cerebellar tonsils to herniate through the foramen magnum^9,10^. The sister, mother, and grandmother of the patient in the present report did not have scaphocephaly. In a large case series, gender differences in the disease severity of XLH were not observed^7,11^; however, the severity and clinical manifestations of XLH patients varied markedly within the same family^12^. Among the family members tested, only the boy presented with sagittal synostosis and a mild Chiari malformation. Congenital forms of craniosynostosis mostly develop in infancy, whereas those caused by metabolic disorders are more common in infants aged ~2 years^10^. Therefore, the younger sister was followed up for symptoms of craniosynostosis.

In summary, we herein described the novel splicing of and a familial mutation in *PHEX*. An early diagnosis facilitates the rapid initiation of proper treatment. Since we detected the same mutation in the younger sister of the patient in our report, she underwent regular follow-ups. We will also carefully monitor symptoms and disease progression in this family in the future.

## Data Availability

The relevant data from this Data Report are hosted at the Human Genome Variation Database at 10.6084/m9.figshare.hgv.2823.
